# Mechanical Behavior of Flexible Fiber Assemblies: Review and Future Perspectives

**DOI:** 10.3390/ma17246042

**Published:** 2024-12-10

**Authors:** Peng Wang, Jiawei Han, Siyuan Wang, Yu Guo

**Affiliations:** 1 Huanjiang Laboratory, Zhuji 311800, China; 12224048@zju.edu.cn (P.W.); hanjw@zju.edu.cn (J.H.); 22124007@zju.edu.cn (S.W.); 2 Department of Engineering Mechanics, Zhejiang University, Hangzhou 310027, China

**Keywords:** granular material, flexible fiber, mechanical behavior, compression, shear flow, gas–fiber two-phase flow

## Abstract

Flexible fibers, such as biomass particles and glass fibers, are critical raw materials in the energy and composites industries. Assemblies of the fibers show strong interlocking, non-Newtonian and compressible flows, intermittent avalanches, and high energy dissipation rates due to their elongation and flexibility. Conventional mechanical theories developed for regular granular materials, such as dry sands and pharmaceutical powders, are often unsuitable for modeling flexible fibers, which exhibit more complex mechanical behaviors. This article provides a comprehensive review of the current state of research on the mechanics of flexible fiber assemblies, focusing on their behavior under compression, shear flow, and gas–fiber two-phase flow processes. Finally, the paper discusses open issues and future directions, highlighting the need for advancements in granular theories to better accommodate the unique characteristics of flexible fibers, and suggesting potential strategies for improving their handling in industrial applications.

## 1. Introduction

Flexible fibers are encountered in many applications, such as biomass materials in the production of biomass fuels, glass fibers in the production of fiber-reinforced composites and 3D printing materials, and wool and hair in the production of textiles. Additionally, applications of flexible fibers are rapidly expanding in emerging industries such as functional sensors [1], medical devices [2], and art exhibitions [3], all of which are expected to contribute significantly to economic growth. Compared to regular hard particles, the flexible fibers have two distinct characteristics: elongated shape and flexibility. The fiber elongation promotes interlocking and entanglement of packed fibers, resulting in very poor flowability. Thus, transport of flexible fibers imposes a challenge on various applications. On the other hand, fiber interlocking enhances shear strength, allowing the formation of self-supporting structures. Fiber beds exhibit remarkable compressibility due to the fiber flexibility. Thus, the microstructures and contact network of the fiber system change subject to the variation of applied loads. A lot of energy can be dissipated in rapid fiber deformation, e.g., bending vibration, due to viscous damping in the material deformation. As a result, flexible fiber systems have high rates of energy dissipation.

Compared to regular granular materials (hard sphere-like particles, for example), the fibers, due to their distinct properties, exhibit more complex mechanical behaviors that are difficult to predict. In this article, we review state of the art and new developments on the mechanics of flexible fiber assemblies and discuss the behaviors in three typical processes: compression, shear flow, and gas–fiber two-phase flow.

## 2. Compression

In the compression process of granular assemblies, the interactions between fiber granules play a crucial role. They bend, twist, or slide against each other upon contact, thereby influencing mechanical responses in the bulk materials. Particularly, the deformation of flexible fibers can better adapt to the shapes and movements of surrounding particles, affecting the microscopic packing structure. Consequently, the assemblies of flexible fibers exhibit distinct compression behaviors from conventional granular materials.

### 2.1. Isotropic Compression

Isotropic compression, in which identical pressures are exerted on the sample in three orthogonal directions, has been widely used in the studies of the mechanical responses and microstructures of granular materials subject to compaction. To quantitatively characterize the structure and robustness of jamming states, two characteristic parameters are frequently used: (i) solid volume fraction

(1) $$\phi =\frac{V_{p}}{V_{s}},$$

defined as the ratio of the volume of all the solid particles $V_{p}$ to the total volume $V_{s}$, for the measurement of particle packing density; (ii) coordination number

(2) $$Z=\frac{2C}{N},$$

defined as the average number of contacting neighbors per particle, for the measurement of mechanical connectivity of the particles in the assembly, where C represents the total number of contacts and N is the total number of particles in the assembly.

**Figure 1:**
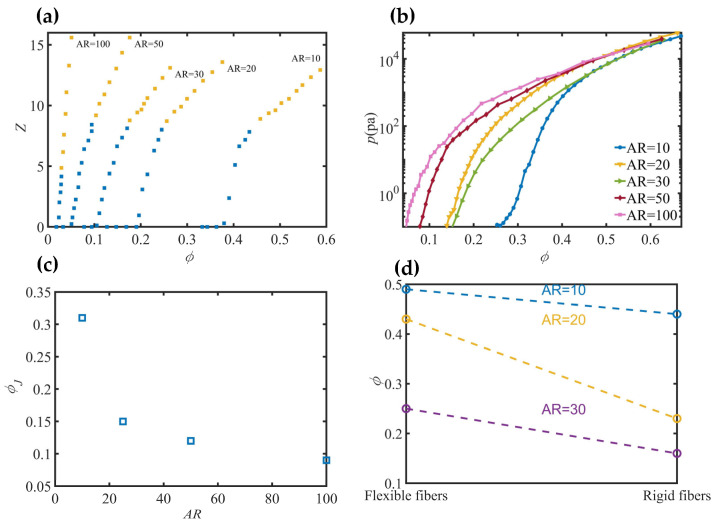
Characteristic parameters for jammed flexible fiber materials: (**a**) coordination number *Z* varying with solid volume fraction ϕ from [4], (**b**) mean pressure p varying with ϕ, (**c**) critical solid volume fraction $\phi_{J}$ varying with fiber aspect ratio *AR* from [6], and (**d**) solid volume fractions ϕ for packed flexible and rigid fibers from [7].

Previous studies primarily focused on the entanglement transition of flexible fibers under isotropic compression [4]. As the compression proceeds, the solid volume fraction of the entire system ϕ gradually increases, and the coordination number Z undergoes two stages of changes. As shown in Figure 1a, with the increase in ϕ, Z initially experiences a rapid growth (blue squares in Figure 1a). At this stage, the fibers aggregate, collide, and rearrange with little deformation. When compressed beyond the critical volume fraction, the rate of increase in Z significantly decreases (yellow squares in Figure 1a), indicating the onset of entanglement, during which the fibers undergo pronounced bending deformations. The critical coordination number $Z_{c}$ for the entanglement transition is smaller for longer fibers than for shorter ones. When the fiber aspect ratio *AR* is less than 50, the critical coordination number $Z_{c}$ is eight, while it decreases to four when *AR* increases to 100.

The packings of various *AR* fibers were further compressed to a large solid volume fraction of ϕ = 0.64 [5]. As shown in Figure 1b, the average pressure p sharply increases with the solid volume fraction ϕ for various fibers at the early stage of compression, and then the pressures for different *AR* fibers tend to collapse onto the same curve. The significant differences for the fibers with different *AR*s at the early stage are due to differences in the natural packing limit and fiber bending deformation. When the fiber packings are compressed to the stage that the fiber bending deformation is difficult, due to the limited void space, the increase in pressure p arises from the enhanced fiber–fiber contacts, causing the collapse of the different pressure curves.

To explore the influence of fiber properties on jamming phase transition, in which the granular system transits from liquid-like to solid-like state, the critical state of the transition is determined as the pressure of the system p achieves a critical level [5,6]. Figure 1c shows the jamming point $\phi_{J}$, defined as the solid volume fraction at the critical state, decreases with increasing fiber aspect ratio *AR* [6]. In addition, the jamming point $\phi_{J}$ increases as the maximum fiber bending deformation increases (i.e., the fiber flexibility increases) [5,6]. The solid volume fractions ϕ of random packings of the flexible and rigid fibers are shown in Figure 1d [7]. The larger flexibility and aspect ratio *AR* lead to larger ϕ due to larger fiber bending.

### 2.2. Uniaxial Compression

A granular sample is compressed in a specified direction with the orthogonal directions constrained by static solid sidewalls, which is known as uniaxial compression. Under such compression, especially subject to cyclic, edometric loading conditions, one can delve deeper into the mechanical behaviors through multiple compression–relaxation cycles.

**Figure 2:**
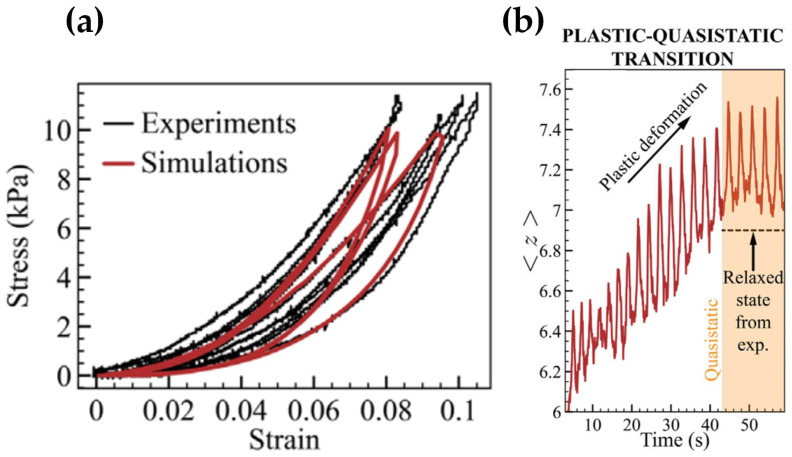
Bulk mechanical and micromechanical responses [8]: (**a**) quasistatic stress–strain cycles, and (**b**) coordination number as a function of time for the initial plastic cycles and the quasistatic regime (highlighted in orange).

Recently, Bhosale et al. [8] studied the uniaxial compression mechanical behavior of a randomly packed sample of 400 bamboo fibers with an aspect ratio of 31 under loading and unloading. In stress–strain curves shown in Figure 2a, two specific mechanical behaviors can be observed: (i) instantaneous plasticity during the initial compression cycle followed by a nonlinear stress–strain curve adopting a repeatable steady-state shape; (ii) finite hysteresis at sufficiently low loading rates, indicating energy dissipation through a non-viscous mechanism. Both behaviors can qualitatively be observed in spherical particles [9] and fibers with an aspect ratio of 1000 [10], though the extent of hysteresis depends on the particle properties [11].

Numerical simulation and three-dimensional Computed Tomography (CT) scan imaging techniques were used to uncover the microscale mechanisms behind the macroscopic observation [8]. As depicted in Figure 2b, during compression the coordination number Z increases during the plastic deformation but exhibits reversible changes at the quasistatic stage. The increase in Z is the microscopic reason for the stiffening of the system during the plastic deformation, and the repeatable steady-state shape of the stress–strain curve is due to the repeatability of Z at the quasistatic stage. The asymmetry of the stresses between loading and unloading was attributed to the sliding contacts of the fibers [8]. Sliding occurred in one direction along the fiber length during loading and returned in the opposite direction during unloading. Thus, the frictional forces at the contacts changed directions between loading and unloading processes, causing the asymmetry of the stress–strain curves. In addition, compression increased the energy stored in the force chains. As a result, the sliding becomes easier during the unloading process as the energy is released.

In uniaxial compression with remarkable fiber deformation and contacts at the large solid volume fractions, the elastic models for fiber–fiber contact and fiber bending deformations can significantly overestimate the loads exerted on the sample of the plastic fibers, and thus it is essential to consider the plasticity of fiber bending and fiber–fiber normal contacts in the DEM simulations to obtain more accurate results [12,13]. Currently, assemblies of plastic fibers are rarely investigated, and the effects of fiber plasticity on the bulk behaviors are not well understood.

### 2.3. Triaxial Compression

Triaxial compression, in which the sample is compressed in one direction and confined with a constant pressure in the directions orthogonal to the compression direction, has been widely used to measure the shear yielding properties of granular materials. Subramanian & Picu [14] numerically studied mechanical behaviors of non-thermal fibers in a non-bonded network under triaxial compression. They found a power-law relationship between stress and volumetric strain, with hysteresis during the loading and unloading processes. After the first loading and unloading cycle, a stable hysteresis loop was established, similar to uniaxial compression. At the early stage of the compression, strain energy was primarily stored in the fibers, due to bending deformation, while at the late stage of compression, a larger amount of strain energy was induced by axial deformation, as limited space existed in the very compacted packing. It was also found that the power-law exponents and energy partition between axial and bending modes depended on the ratio of fiber bending to axial stiffness.

**Figure 3:**
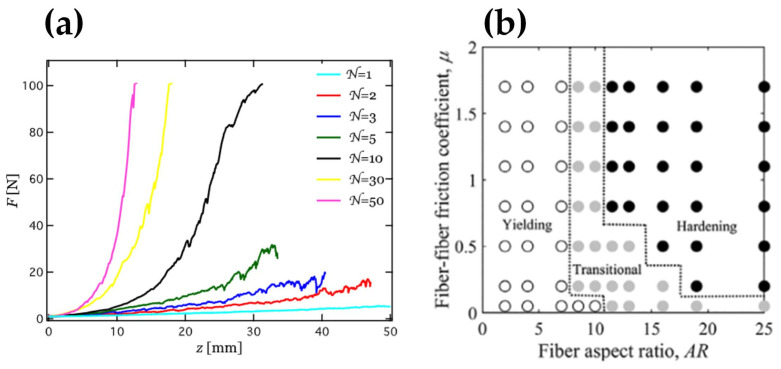
(**a**) Resistance force F as a function of indentation depth z, for granular chain assemblies with various numbers *N* of beads per chain [16]. (**b**) Phase diagrams of yielding, transitional, and hardening regimes based on the fiber aspect ratio, *AR*, and fiber–fiber friction coefficient μ for confining pressure $p_{con}$= 10 kPa [18]. The circle symbols in figure are the simulation results.

The fiber aspect ratio *AR* has a significant impact on triaxial compressions. A large number of monodisperse granular chains (each chain composed of *N* connected steel beads) were placed in a floppy container and axially compressed [15,16]. The results showed that the systems of the shorter chains (smaller *N*) yielded with the shear stresses capped by an upper limit, similar to those of spherical particles (*N* = 1) [17]. Longer granular chains (larger *N*) formed loop structures and exhibited strain hardening, for which the effective stiffness of the system increased with strain (Figure 3a). The X-ray tomography measurements revealed that space-spanning clusters of entangled long chains were generated in the strain-hardened granular packings. To quantitatively predict the relationship between indentation depth z and resistance force F in the compression, an interlocking model based on frictional self-amplification effects and polymer physics was proposed to elucidate the strain hardening behavior in the form of

(3) $$F\sim \mu \sqrt{N}\phi^{11/8}z/b$$

where μ represents the friction coefficient between two elementary beads, b denotes bead diameter, and ϕ indicates the solid volume fraction of the granular chains.

These granular chains composed of steel beads [15,16] typically had high flexibility, and long chains could also result in high packing densities. Guo et al. [18] comprehensively studied the effects of fiber properties, including fiber aspect ratio, friction coefficient, and flexibility, on strain hardening [18]. Based on the results of triaxial compression tests, a phase diagram was obtained in Figure 3b, identifying three different mechanical behaviors: yielding, transition, and hardening. As the friction coefficient increased, the fiber aspect ratio *AR* associated with strain hardening decreased. They also observed that a decrease in fiber flexibility led to a decrease in the fiber aspect ratio associated with strain hardening, affecting the boundaries of the three regimes [18].

Additionally, it was found that the yield strength of the spherical particle systems was increased by adding some flexible fibers to the systems [19], and this fiber-reinforcement effect could even lead to strain hardening. Further works have been devoted to the mechanics of assemblies made of more complex objects, such as stars, or U- and Z-shaped particles [20,21,22,23]. It was found that the fiber packings could bear the tensile force due to the fiber entanglement, and the increase in the number of force chains was a key factor in enhancing the yield strength [20]. It was also observed that fiber orientation affects compression properties, with fibers oriented perpendicular to the load direction had better dynamic properties than parallel to the load direction [24].

## 3. Shear Flow

**Figure 4:**
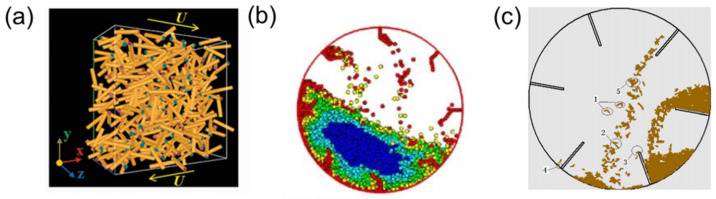
Fiber shear flow schematics: (**a**) elongated rods at plane shear, where the blue and red dots indicate the two end faces of the cylinder. [27]; (**b**) heat transfer of flexible fibers in a rotating drum, with particles of the same color representing those at the same temperature [28]; (**c**) flexible filamentous particles in a rotary dryer with flights [29].

Granular materials undergoing shear flow experience external forces (e.g., gravitational force, magnetic force, etc.) and contact forces from neighboring particles, displaying mechanical behaviors resembling those of solids or liquids. They can undergo transitions between these states under specific conditions. The shear flow of fibers is prevalent in natural phenomena and industrial processes like biomass material handling. These processes involve multiple scales, including the nanoscale, which adds significant complexity to the shear flow behavior. Specifically, the mechanical properties at the nanoscale—such as stiffness, elasticity, and adhesion—are crucial in determining fiber durability. This durability, in turn, is essential for the performance and effectiveness of fibers in a wide range of industrial applications [25,26]. Figure 4 shows the numerical simulation results of planar shear flows of rod-like fibers [27], heat transfer of flexible fibers in a rotating drum [28], and filamentous particles in a rotary dryer with flights [29], respectively. In these processes, denser, compressible flows with smaller solid-phase stresses are observed for the more flexible fibers. The effects of fiber properties on the flows are discussed in this section.

### 3.1. Overview of Granular Flow

In dilute and moderately dense granular flows, particle–particle collisions are binary and instantaneous. Based on this fact, a continuum method, granular kinetic theory [30], is introduced. It describes the solid-phase stress tensor of a bulk system ($\sigma_{tot}$) as the sum of a kinetic or streaming component ($\sigma_{kin}$) and a collisional component ($\sigma_{col}$):

(4) $$\sigma_{kin}=\rho \phi \langle CC\rangle$$

(5) $$\sigma_{col}=\langle F_{c}l_{c}\rangle$$

in which ρ is the material density of particles and ϕ is the solid volume fraction. The angle brackets 〈 〉 represent an average over time and space, $C=c_{i}-\langle c\rangle$ is the fluctuating velocity of particle *I*, $F_{c}$ is the contact force exerted on particle *i* by particle *j*, and $l_{c}$ is the vector connecting the mass centers from particle *j* to particle *i*.

In dense granular flows, prolonged collisions become prevalent with contact stiffness emerging as a significant factor [31,32]. Consequently, the conventional kinetic theory of gases, which assumes instantaneous binary molecular collisions, proves inadequate for capturing the characteristics of dense flows. To address this problem, frictional contact should be accounted for as a contributor to granular stresses [33,34] Campbell et al. [31,32,35] categorized the granular flows into two major regimes, namely inertial and elastic regimes, based on dimensionless stress, $\sigma d_{p}/k$, and dimensionless stiffness, $k/(\rho d_{p}^{3}\gamma^{2})$, in which $d_{p}$ represents the particle diameter, *γ* is the shear rate, and k is the particle–particle contact stiffness. In the inertial regime, characterized by predominantly binary and transient contacts, the stresses are independent of the contact stiffness k and they are proportional to $\gamma^{2}$. The elastic regime, dominated by dense networks of long-lasting contact force chains, can be further divided into two sub-regimes: elastic-quasi-static and elastic-inertial. In the elastic-quasi-static regime, the stresses are independent of *γ* and increase linearly with contact stiffness k. At higher shear rates, a transition may occur from the elastic-quasi-static regime to the elastic-inertial regime, where stresses exhibit a linear relationship with the product of shear rate and root square of contact stiffness, $\gamma \sqrt{k}$. Jop et al. [36] demonstrated through experimental and theoretical studies that the packing density ϕ and bulk friction coefficient μ are functions of the inertial number ($I=\gamma d_{p}\sqrt{\rho /p}$), where $d_{p}\sqrt{\rho /p}$ represents the pressure-induced inertial timescale, and $\gamma^{-1}$ is the timescale of deformation. The μ−I correlation can be written as

(6) $$\mu =\mu_{s}+\frac{\mu_{m}-\mu_{s}}{1+I_{0}/I}$$

where the model parameters $\mu_{s}$, $\mu_{m}$, and $I_{0}$ depend on the particle characteristics (friction, shape, polydispersity, etc.).

### 3.2. Effect of Fiber Aspect Ratio

**Figure 5:**
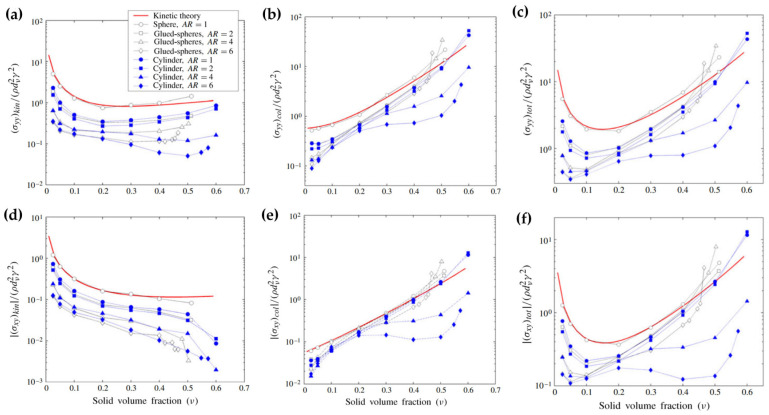
Solid-phase stress results of rigid rod-like particle systems (cylindrical or glued spheres) with different aspect ratios and no friction [37]. (**a**) Kinetic components of the normalized normal stresses, (**b**) collisional component of the normalized normal stresses, (**c**) total stress of the normalized normal stresses, (**d**) kinetic components of the shear stresses, (**e**) collisional component of the shear stresses, and (**f**) total stress of the shear stresses as a function of solid volume fraction ϕ.

The aspect ratio of fibers *AR* has a profound influence on the shear flows. Figure 5 depicts the solid-phase stress results of sheared frictionless fiber systems at various solid volume fractions with different fiber aspect ratios [37]. The red solid line represents the prediction of granular kinetic theory, which matches well with the numerical results for spherical particles (*AR* = 1) at the solid volume fractions smaller than 0.4. The kinetic theory can be reliably applied to smooth spheres in dilute and moderately dense systems. However, for dense granular flows and non-spherical particles with complex shapes, significant deviation exists. This is because the assumption of binary, transient collisions in the kinetic theory is violated in the dense flows, where long-lasting collisions dominate. The shapes of particles affect collisional frequency, contact forces, and microscopic contact structures, influencing the solid-phase stresses.

Based on the dependence of the solid phase stresses on the solid volume fraction, frictionless fiber flows can be divided into three regimes: dilute, dense, and intermediate. In the dilute regime, the kinetic stresses predominate with the momentum mainly transferred through particle motion. In the dense regime, the collisional stresses dominate with the momentum transferred through interparticle collisions. The third is the intermediate regime, which transits between the dilute and dense regimes. In this regime, the kinetic and collisional components are comparable, resulting in the decrease-to-increase transition in the total stresses. It can be observed from Figure 5 that as the particle aspect ratio *AR* increases, the solid-phase stress decreases. This is because the particles align with their major/long axes at a small angle to the direction of flow. This particle alignment reduces interactions between particles and their neighbors.

**Figure 6:**
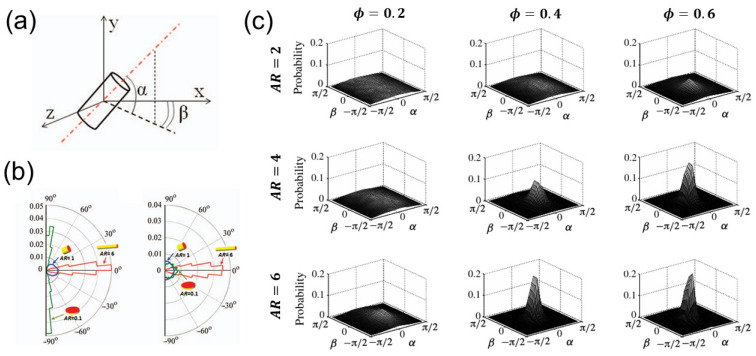
The schematic diagrams and probability distributions for inclination angle α and azimuthal angle β: (**a**) schematic diagram of inclination angle α and azimuthal angle β, (**b**) probability distributions for inclination angle α and azimuthal angle β for the fibers with aspect ratios *AR* = 0.1, 1, and 6 [27]; (**c**) probability distributions for inclination angle α and azimuthal angle β for fibers with different particle aspect ratios *AR* and different solid volume fractions ϕ [37].

Figure 6a introduces the inclination angle α and azimuthal angle β of a cylindrical particle [27]. In Figure 6b, the cylindrical particles with an aspect ratio of *AR* = 1 exhibit a uniform probability distribution of inclination angle α. In contrast, the flatter particles with an aspect ratio of 0.1 or longer particles with an aspect ratio of 6 tend to align at 0 degrees or 90 degrees. Moreover, the particles with aspect ratios of 0.1 or 6 exhibit a higher fraction of azimuthal angle β at 0 degrees compared to particles with an aspect ratio of 1. The alignment is enhanced with flatter or more elongated particles. In addition, as shown in Figure 6c, with increasing solid volume fraction, particle alignment becomes more pronounced. The effective particle projected area on the plane perpendicular to the flow direction decreases with increasing particle aspect ratio at high solid volume fraction, affecting stresses by influencing the likelihood of particle collisions.

However, for the shearing flows of frictional fibers, as the fiber aspect ratio increases, regular particle alignment does not occur, due to the stronger interaction. Thus, the number of contacts between particles increases, facilitating the formation of complex force chains, leading to an increase in solid-phase stress [38]. In fiber shear flows where liquid-bridge force exist, fiber aggregation occurs due to the presence of liquid viscous force, with the aggregate size increasing with increasing particle aspect ratio [39].

### 3.3. Effects of Friction and Particle Surface Roughness

Interparticle friction plays a crucial role in determining the flow pattern, particle alignment, and stress distribution. Unlike smooth laminar-like flows observed with frictionless particles, the presence of friction causes elongated particles to rotate in shear flows, leading to an increase in particle–particle interactions. The rotational motions of the particles result in fluctuating flow streams.

**Figure 7:**
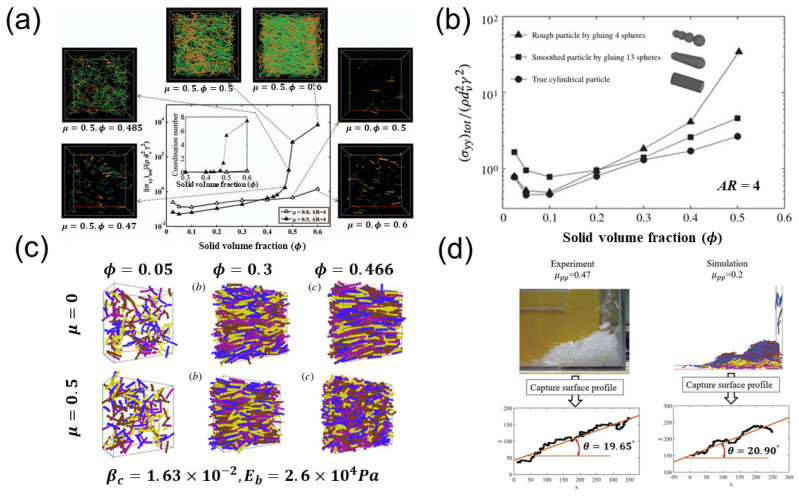
(**a**) Variation of normalized shear stress with solid volume fraction for particles with and without friction. The surrounding images show snapshots of force chains at the specified solid volume fractions [27]. (**b**) Comparison of stresses with rough glued-sphere, smooth glued-sphere particles, and cylindrical particles [37]. (**c**) Snapshots from shear flows of flexible fibers at different solid volume fractions with or without friction [38]. (**d**) DEM and experimental results of angles of repose of the wet fibers [39].

In dense shear flows of frictional particles, a slight increase in solid volume fraction leads to a sharp increase in stresses, causing the formation of a dense network of force chains. Consequently, the system transitions from a fluid-like state to a solid-like state, known as jamming, as depicted in Figure 7a. This phenomenon can occur at lower solid volume fractions for flatter or longer particles [27].

The surface roughness of the particles also plays a significant role in dense flows. As the particles slide past the surfaces of their neighbors, irregularly shaped agglomerate particles are prone to more collisions and stronger interlocking compared to smooth cylindrical particles. Therefore, as the particle surfaces become more irregular or rough, the shear and normal stresses surge at higher solid volume fractions [37], as illustrated in Figure 7b. The comparison between the glued-sphere and cylindrical particles in Figure 5 also demonstrates the increased solid-phase stresses due to particle surface roughness.

The shear flow patterns are influenced by interparticle friction. As shown in Figure 7c, significant particle alignment occurs for the frictionless fibers, while large fiber bending deformation is observed fibers with the friction coefficient of 0.5 [38]. Due to the surface roughness of the numerical particle model, smaller friction coefficients are required in the numerical simulations than in the experiments in order to achieve a similar angle of repose [39], as shown in Figure 7d.

### 3.4. Effect of Fiber Flexibility

**Figure 8:**
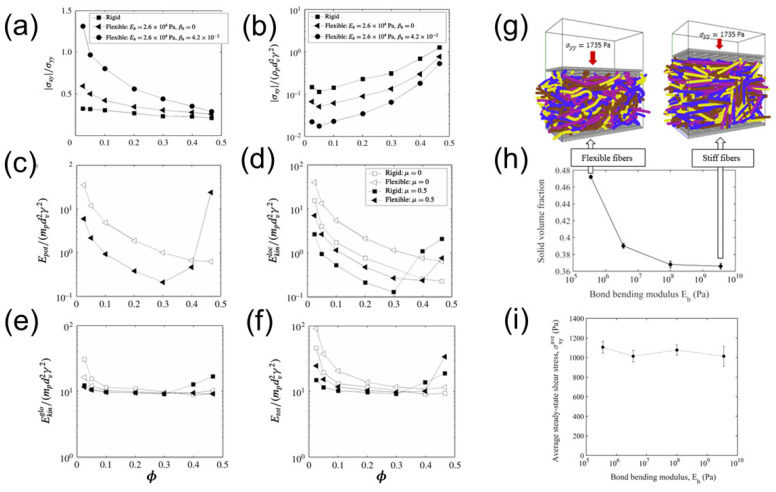
(**a**) The apparent friction coefficient and (**b**) normalized shear stress as a function of solid volume fraction for rigid and flexible fibers without friction. The average mechanical energies, including (**c**) elastic potential energy (PE), (**d**) local kinetic energy (LKE), (**e**) global kinetic energy (GKE), and (**f**) total mechanical energy (TME), per fiber as a function of solid volume fraction in the shear flows of rigid and flexible fibers with and without friction. Panels (**c**–**g**) shard a common legend [38]. (**g**) Snapshots of the fibers with different flexibility. (**h**) Solid volume fraction and (**i**) average steady-state shear stress as a function of bond bending modulus at a normal stress of $\sigma_{yy}=1735\;Pa$ [40].

According to kinetic theories for granular flow [30], the shear normal stress, $\sigma_{yy}$, is proportional to the particle temperature *T*, while the shear tangential stress, $\sigma_{xy}$, is proportional to $\sqrt{T}$. Therefore, the apparent friction coefficient, $\left|\sigma_{xy}\right|/\sigma_{yy}$, is inversely proportional to $\sqrt{T}$. Based on the relationship between solid-phase stress and coefficient of restitution, the shear stress should be positively correlated with the coefficient of restitution, while the apparent friction coefficient $\left|\sigma_{xy}\right|/\sigma_{yy}$ should be negatively correlated with the coefficient of restitution. As a result, larger apparent friction coefficients $\left|\sigma_{xy}\right|/\sigma_{yy}$ are obtained for the more flexible fibers [38], which have smaller coefficients of restitution, as shown in Figure 8a, and smaller shear stresses are observed for the more flexible fibers (Figure 8b).

The total mechanical energy (TME) includes kinetic energy ($E_{kin}$) and elastic potential energy (PE). The kinetic energy of a fiber, $E_{kin}$, can be decomposed into global kinetic energy (GKE), $E_{kin}^{glo}$, and local kinetic energy (LKE),$\;E_{kin}^{loc}$. GKE is caused by the translation of the fiber. LKE describes the kinetic energy relative to the mass center of the fiber, and it is generated by the rotation and vibration of the fiber. The elastic potential energy (PE) of a fiber is generated due to the elastic deformation of bonds. They have the forms

(7) $$E_{kin}=E_{kin}^{glo}+E_{kin}^{loc}$$

(8) $$E_{kin}^{glo}=\frac{1}{2}m_{p}v_{O}^{2}$$

(9) $$E_{kin}^{loc}=\frac{1}{2}\sum_{i=1}^{N_{s}}m_{si}{\left(v_{si}^{r}\right)}^{2}+\frac{1}{2}\sum_{i=1}^{N_{s}}J_{si}{\left(\omega_{si}\right)}^{2}$$

(10) $$E_{pot}=\sum_{i=1}^{N_{b}}\left[\frac{1}{2}\frac{E_{b}A}{l_{b}}{\left(\delta_{ni}^{b}\right)}^{2}+\frac{1}{2}\frac{G_{b}A}{l_{b}}{\left(\delta_{ti}^{b}\right)}^{2}+\frac{1}{2}\frac{G_{b}I_{p}}{l_{b}}{\left(\theta_{ni}^{b}\right)}^{2}+\frac{1}{2}\frac{G_{b}I}{l_{b}}{\left(\theta_{ti}^{b}\right)}^{2}\right]$$

in which, $m_{p}$ is total mass and $v_{O}$ is the translational velocity of the mass center of a fiber, $N_{s}$ is the number of constituent spheres in a fiber, $v_{si}^{r}$ is the relative velocity of the constituent sphere *i* of mass $m_{si}$ to the center of mass of the fiber, and $J_{si}$ and $\omega_{si}$ are the moment of inertia and angular velocity (in the global frame of reference) respectively of the constituent sphere *i*. $N_{b}$ is the number of bonds in a fiber. In addition, $\delta_{ni}^{b}$, $\delta_{ti}^{b}$, $\theta_{ni}^{b}$, and $\theta_{ti}^{b}$ represent the current normal, shear, torsional and bending deformations, respectively, of the bond *i*.

From the energy perspective, as shown in Figure 8c–f, at lower solid volume fractions, the rearrangement of fibers reduces the PE and LKE of flexible fibers. At higher solid volume fractions, enhanced fiber–fiber contacts result in larger fiber deformations, greater PE, and stronger fiber vibrations, and thus a larger energy of the system. Compared to frictionless flexible fibers, an increase in the solid volume fraction leads to significant deformation of the fibers with friction, causing the formation of complex force chain structures, higher PE, and larger solid-phase stresses.

Figure 8g depicts the planar shear flows of flexible fibers under a fixed normal stress [40]. According to Figure 8h,i, an increase in fiber bending modulus leads to a reduction in fiber deformation and solid volume fraction, while it has no impact on the shear stress under the same normal stress. Moreover, greater fiber flexibility results in a higher average coordination number (*CN*), promoting agglomeration in flows of wet flexible fibers [39] and heat transfer in a rotating drum [28].

### 3.5. Effect of the Shapes of the Fibers at Rest

**Figure 9:**
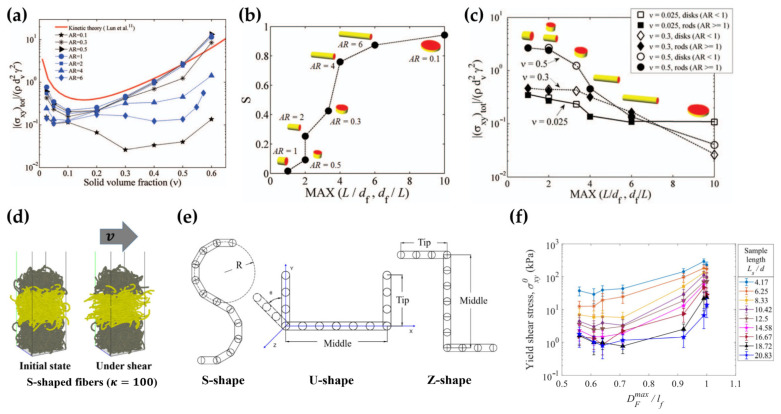
(**a**) Normalized shear stress as a function of solid volume fraction for various particle aspect ratios. (**b**) Variation of order parameter *S* with the maximum dimensional ratio of ($L/d_{f}\;and\;d_{f}/L$) for dense flows at the solid volume fraction of ϕ=0.5. (**c**) Normalized shear stresses as a function of the maximum value of $L/d_{f}\;and\;d_{f}/L$ at different solid volume fractions [27]. (**d**) Numerical models of shear tests of S-shaped fibers, the gray particles represent stationary particles, while the yellow particles indicate moving particles. (**e**) Sketches of S-shaped, U-shaped, and Z-shaped fibers. (**f**) Yield shear stress versus normalized maximum Feret diameter of a fiber $D_{F}^{max}/l_{f}$ for various sample lengths [43].

In frictionless rod-like or disk-like particle systems [27], the solid-phase stresses decrease as the particle shape deviates from a sphere, as illustrated in Figure 9a. The maximum ratio between the cylindrical particle length, *L,* and the circular face diameter $d_{f}$ ($L/d_{f}\;or\;d_{f}/L$), is identified as a significant factor in determining the solid-phase stresses in plane shear flows. In steady dense flows at high solid volume fractions, non-spherical particles tend to align preferentially, orienting their largest dimensions (radial dimension for disks and axial dimension for rods) at a small inclination angle with respect to the flow direction. Particle orientation can be assessed through the diagonalization of the symmetric traceless order tensor ***Q*** [41,42]:

(11) $$Q_{ij}=\frac{3}{2N}\sum_{n=1}^{N}\left[l_{i}^{n}l_{j}^{n}-\frac{1}{3}\delta_{ij}\right]$$.

The order parameter, denoted *S*, corresponds to the largest eigenvalue of ***Q***, indicating the extent of particle alignment. A unit value of *S* suggests complete alignment of all particles along a specific direction, whereas a zero value of *S* indicates uniform distribution of the particle orientations in space. It was found the particle alignment is enhanced as the particles become flatter or more elongated, as depicted in Figure 9b. Due to the alignment, particle–particle interaction is minimized, leading to a reduction in the stresses and apparent friction coefficients (Figure 9c).

The S-shaped, U-shaped, and Z-shaped flexible fibers have been examined in the shear tests [43] as shown in Figure 9d,e. Reduction in yield shear stresses and coordination numbers was observed as the fiber curvature *κ* increases for the S-shaped fibers. Additionally, U-shaped and Z-shaped fibers exhibited smaller yield shear stresses and coordination numbers. For all the fibers considered, the yield shear stress was determined by a maximum Feret diameter $D_{F}^{max}$, representing the largest dimension of a fiber between two parallel tangential lines. The yield shear stress increased with increasing maximum Feret diameter of the fibers, as shown in Figure 9f.

## 4. Gas–Fiber Two-Phase Flow

Multiphase flows with fibrous particles have attracted significant attention due to the complexity in fiber motion and fluid-fiber interaction. Previous research in this field has focused on sparse fiber suspensions with a low concentration of solid fibers, which have been comprehensively reviewed in [44] and will not be further elaborated here. In this work, we will review the gas–solid two-phase flows with high concentrations of the fibers. The experimental and numerical methodologies were employed in these investigations. For the numerical studies, Claeys et al. [45,46] used ellipsoidal models to represent elongated fibers in Stokes flows. Their method applies Stokesian dynamics to simulate interactions between particles, with improved efficiency using the Ewald summation technique. This approach allows for accurate predictions of the transport properties of ellipsoidal structures. Nan et al. [47], adopting the multi-sphere model [48], treated the fibers as rod-like particles in their simulations to study fluidization behavior. By combining the discrete element method (DEM) and computational fluid dynamics (CFD), they found that the particle aspect ratio significantly affects the fluidization dynamics, including bed permeability, coordination number, and particle interactions. Their results emphasize the importance of considering particle shape, particularly the aspect ratio, when modeling fiber behavior in fluidized systems. Although these models could mimic the particle shape, they overlooked the particle deformation characteristics inherent to fibers. To address this limitation, The flexible fiber models based on virtual, deformable bonds were proposed [49] to simulate the fibers that experience remarkable deformation. In the following sections, we will discuss the fluidization, pneumatic transport, segregation, and clustering behaviors of flexible fibers at high concentrations.

### 4.1. Fluidization

Fluidization technology is widely applied in various industries for processing and handling of granular materials, due to its efficient mass and heat transfer capabilities. Compared to the fluidization of rigid, round particles, the fluidization of elongated, flexible fibers has been much less investigated. The limited research work suggests that the fiber elongation and flexibility played critical roles in determining the characteristics of the fluidized beds.

**Figure 10:**
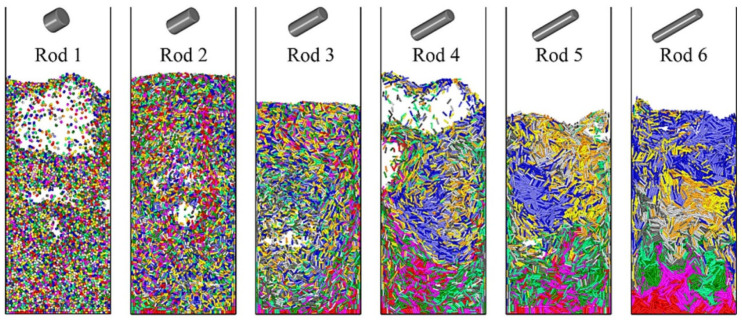
Snapshots of fluidized rod-like particles [50].

Ma et al. [50] employed a coupled approach of DEM and Computational Fluid Dynamics (CFD) to explore the flow behaviors of ellipsoidal particles with aspect ratios ranging from *AR* = 1 to 6 within fluidized beds (Figure 10). It is found that the minimum fluidization velocity $U_{mf}$ increased with *AR*, attributed to drag forces and interlocking effects among the particles. Additionally, both the bed voidage fraction and mean projected face area of the rod-shaped particle increase with the increase in *AR* in the packed bed. When *AR* approaches 1, the rod-like particles more easily obtain kinetic energy from the gas flow, exhibiting vigorous fluidization behavior. As a result, higher mixing rates and more frequent bubble generation are observed. Moreover, the influences of the side walls on fiber orientation cannot be ignored, as the rod-like particles with higher aspect ratios tend to align parallel to the wall surface near the wall. Similar effects of the particle aspect ratio on minimum fluidization velocity, fluidization patterns, and particle orientation were reported in Nan et al. [47], in which a multi-sphere model was used to simulate the rod-like particles.

The fluidization of dry flexible fibers was numerically investigated by Guo et al. [51]. It is found that as the fiber aspect ratio *AR* increases from 1 to 2, the minimum fluidization velocity $U_{mf}$ decreases due to the increased average projected surface area of fibers within the packed bed. However, as *AR* increases from 2 to 6, $U_{mf}$ increases due to the higher porosity. The solid mixing rates for different *AR*s can collapse as the superficial gas velocity is normalized by the minimum fluidization velocities, i.e., $U/U_{mf}$. In addition, fiber flexibility has a remarkable impact on the fluidization characteristics. Larger flexibility is associated with more pronounced fiber deformation, which reduces the porosity of the packed fiber bed and consequently decreases the minimum fluidization velocity. The solid mixing rates can collapse as a function of the normalized minimum fluidization velocity $U/U_{mf}$ for various fiber flexibilities. The fluidization of wet flexible fibers considering cohesive, liquid-bridge contact forces was explored in [52], and the effects of cohesion on minimum fluidization velocity, solid mixing rate, and clustering were discussed.

In summary, the fluidization of flexible fibers is influenced by a combination of the particle aspect ratio, fiber flexibility, and cohesive forces. These factors must be carefully considered when modeling and designing the fluidization processes of flexible fibers in industrial applications, as they significantly impact efficiency and performance.

### 4.2. Segregation

**Figure 11:**
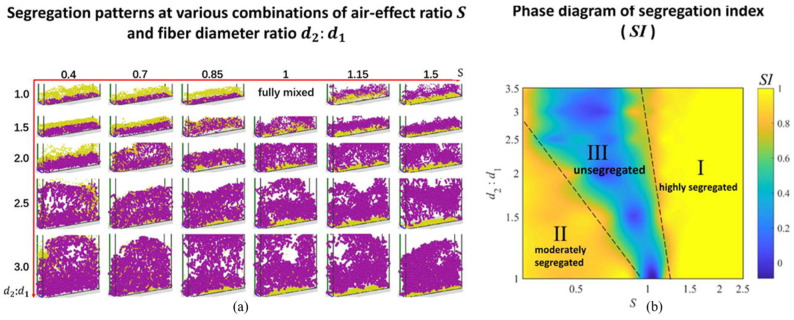
(**a**) Snapshot of segregation results in a binary fiber mixed bed where yellow and purple represent particles with different diameters (**b**) segregation phase diagram [54].

Segregation is referred to as the process in which particles of similar properties (such as size, density, or shape) accumulate in one part of the system, and it has been extensively investigated. However, the segregation of flexible fibers is much less explored. Zhang et al. [53] found that particle shape significantly affected segregation in the fluidization of ternary mixtures of spheres, cylinders, and rod-like particles. Focusing on the flexible fibers, Wang et al. [54] systematically analyzed the influences of fiber density and diameter on the extent of segregation in a fluidized bed of binary fibers. They found that segregation is induced by an air entrainment effect due to air drag difference and a sifting effect due to particle size difference. To quantify the influence of air entrainment, they introduced an air-effect ratio, which depends on the fiber density ratio and diameter ratio. Using DEM–CFD simulations, they showed that the extent of segregation is determined by the interplay between these two effects. Specifically, the air-effect ratio and fiber diameter ratio were used to develop a phase diagram that predicts the extent of segregation in fluidized beds of binary fibers (Figure 11). This approach provides valuable insights into the dynamics of gas–fiber two-phase flows by highlighting the significant roles played by fiber properties and their interactions with the surrounding gas.

### 4.3. Pneumatic Conveying

**Figure 12:**
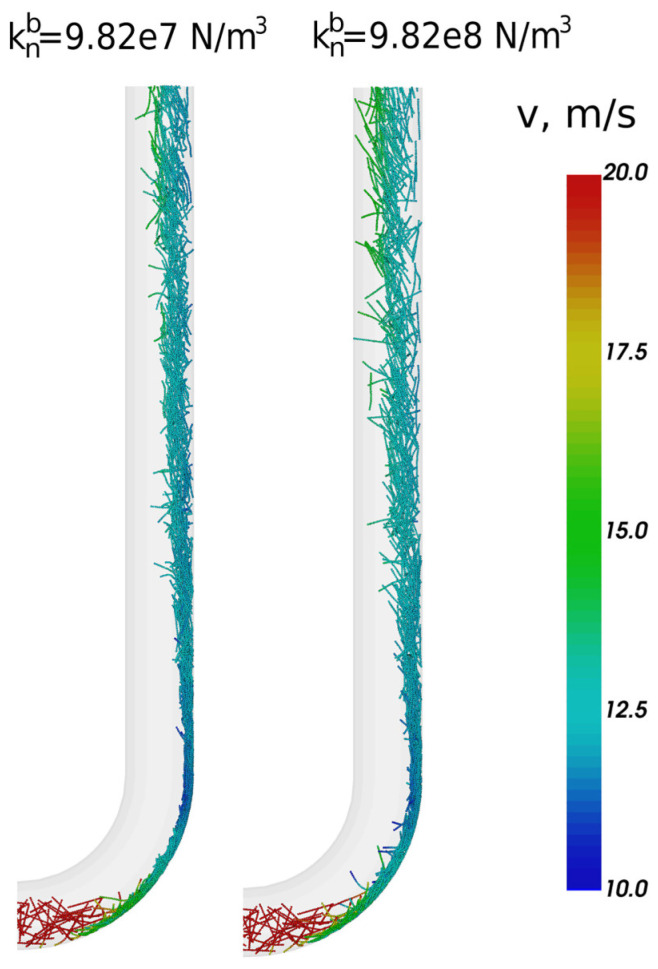
Fibers in the bend and vertical pipe section at time 0.5 s colored by particle velocity, under different bonding stiffnesses [55].

Pneumatic conveying transport of flexible elongated particles through a pipe bend was analyzed using the DEM–CFD by Markauskas et al. [55], as shown in Figure 12. The influences of fiber stiffness, bond damping, fiber mass flow rate, fiber length, and superficial gas velocity on the flow behaviors were discussed. It was found that the trajectories of the fibers and pressure drop within the vertical pipe section were dependent on the fiber stiffness, and rigid fibers exhibited higher flow rates than flexible ones. Furthermore, bond damping coefficients had important impacts on fiber deformation, fiber–fiber contacts, and fiber–wall contacts, and thus influenced fiber velocities and overall transport results.

**Figure 13:**
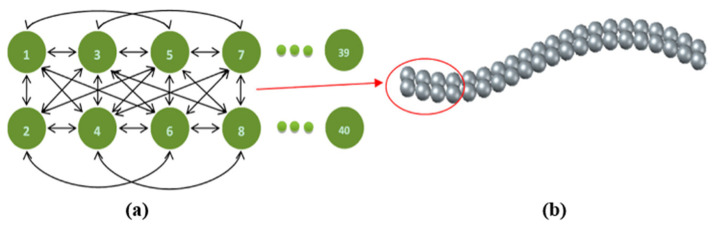
Illustration of a flexible ribbon chain model. (**a**) Schematic diagram of the bond potential of the ribbon shaped particle and (**b**) flexible ribbon chain [56].

Huang et al. [56] numerically studied the pneumatic transport of flexible particles in a riser. In the numerical method, a flexible ribbon chain was generated by connecting elemental spheres using the bonding potential between adjacent elemental spheres, as illustrated in Figure 13. The disturbances, deformations, and clustering near the wall of the fibers were obtained in the simulations, consistent with experimental observations. It was found that the gas velocity had an effect on the axial distributions of the flexible fibers in the riser: the concentration difference between the bottom and top of the riser increased with increasing superficial gas velocity.

Wu et al. [57,58] experimentally and numerically investigated the dynamics of flexible fibers in a riser using an improved Particle Image Velocimetry (PIV) method and a DEM–CFD approach. By selecting a number of characteristic points on individual flexible fibers in the experiments, they managed to track the movement and deformation of the fibers. A higher portion of particles undergoing translational motion indicates a better flow consistency. As the fiber mass flow rate increases, the portion of translational fibers decreases. In the numerical simulations, the flexibility of cut tobacco particles was modeled using a double-stranded particle chain model. The concentration of fibers at the bottom of the riser obtained from the simulations was slightly lower than that in the experiments at lower superficial gas velocities, while the time-averaged radial concentration and velocity distribution of the fibers agree well with the experimental data.

### 4.4. Clustering

**Figure 14:**
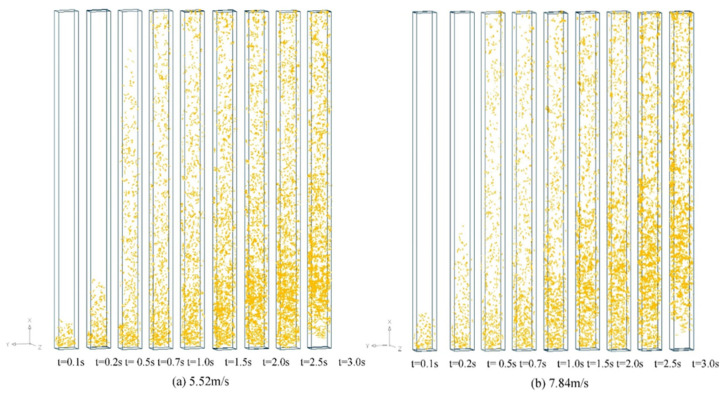
Spatial distributions of fibers in the riser at the superficial gas velocities of (**a**) 5.52 m/s and (**b**) 7.84 m/s [59].

Clustering of the granular materials is frequently encountered in the liquid–particle or gas–particle two-phase flows. Previous studies mainly focused on the clustering of spherical particle systems in the fluidization. According to the open literature that we have noticed, Geng et al. [59] was the first to investigate the distributions and clusters of the flexible fibers in a fluidized bed dryer riser using a numerical method. The results indicated that the fibers tended to be dilute in the center of the riser and dense near the walls. The formation of the clusters in the riser led to an unstable flow, exaggerating the uneven distribution. As illustrated in Figure 14, an increase in the superficial gas flow rate reduced the average residence time of fibers in the riser, promoting a more uniform fiber distribution. However, as the mass flow rate of the fibers increases, the particle distribution becomes more uneven, and the fibers tend to aggregate.

Wu et al. [60] further investigated the distributions and clustering of the flexible fibers in a cold fluidized riser through experiments, considering the effects of fiber length and moisture content on the clustering. It was found that as fiber length and moisture content increased, the cluster size tended to increase. It is worth noting that the changes in fiber moisture content altered the fiber’s flexibility and adhesion, which was distinct from the aggregation caused by the liquid-bridge effect in the wet spherical particle system. Based on extensive experimental data, Wu et al. [60] proposed empirical correlations to describe the average cluster diameter and cluster frequency, providing a valuable reference for subsequent studies on the fiber clusters. Du et al. [61] focused on the uniform distribution of the biomass fibers in the riser in the pneumatic conveying experiments. By examining velocity distributions of fibers under different superficial gas velocities and fiber flow rates, they identified the main fields of fiber aggregation and the reasons for uneven fiber movement. It was also found that fibers near the riser walls moved significantly slower than those in the center, leading to a recirculation of the fibers. Higher superficial gas velocities could improve the uniformity and stability of the airflow, reducing local velocity fluctuations and unevenness and resulting in a more uniform fiber distribution. In addition, the fiber mass flow rate allows fibers to disperse within the riser, enhancing the uniformity of particle distribution.

**Figure 15:**
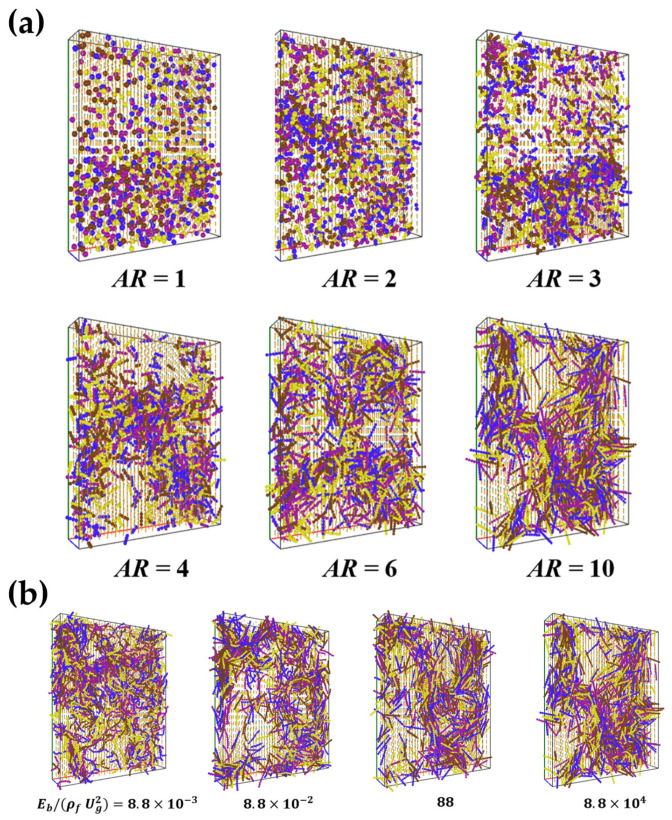
Snapshots of fiber clusters with various (**a**) fiber aspect ratios (AR) and (**b**) dimensionless bending moduli ($E_{b}/\rho_{f}U_{g}^{2}$) in a riser flow with the superficial gas velocity ***U****_g_* = 10 m/s. Average solid volume fraction is *ϕ_a_* = 0.0758. The fibers are colored differently for visualization purposes [62].

Unlike the analyses of fiber distributions in the risers, Xu et al. [62] investigated the effects of the fiber aspect ratio, flexibility, and contact damping of flexible fibers on the clustering in a fluidized bed (Figure 15). The uneven spatial distribution of the fibers was quantified using a heterogeneity index *H*. The strongest fiber clusters, indicated by the average heterogeneity index *H_a_*, occurred at the critical superficial gas velocities when the fibers transited from the downward to the upward flow. For an upward flow, the heterogeneous index *H_a_* decreased with increasing superficial gas velocity until it reached a saturated state. The heterogeneity index *H_a_* decreased with increasing solid volume distribution and increased with the size of the fluidized zone and the number of fibers, but it is insensitive to the shape of the fluidized zone. Since clusters formed by fibers with a large aspect ratio have a lower packing density, the number and size of clusters increase as the fiber diameter ratio increases while the homogeneity index *H_a_* decreases. As the fiber flexibility increases (i.e., the dimensionless bending modulus $E_{b}/\rho_{f}U_{g}^{2}$ decreases), the homogeneity index *H_a_* increases, and the clusters arrange more tightly. Additionally, the effect of fiber flexibility on clusters becomes more significant at larger aspect ratios. From an energetic perspective, an increase in contact damping between colliding fibers leads to increased energy dissipation rates, resulting in a surge in cluster size and number.

## 5. Open Issues and Outlook

Granular flows of popular particles (dry sands and pharmaceutical powders, for example) can be well described by the kinetic theory [30] for dilute flows and by the inertial number I-based theory [36] for dense flows. The existing kinetic theory [30] is not applicable to the shear flows of the fibers [38]. Thus, in future studies, the kinetic theory may need to be modified in order to consider the combined effects of fiber elongation and flexibility on interparticle contacts and collisional dissipation rates [63,64]. It is still an open question whether the inertial number I-based theory [36] can be used to describe the dense flows of the flexible fibers. If the form of Equation (3) can depict flow characteristics of the dense fiber flows under certain conditions, the dependence of the model parameters $\mu_{s}$, $\mu_{m}$, and $I_{0}$ on the fiber aspect ratio and bending elastic modulus needs to be determined. In summary, flexible fiber flows are hard to predict due to a lack of a reliable model of rheology.

Polydisperse systems involving the fibers of different diameters, different lengths, and different bending moduli impose greater challenges on the understanding of fiber flows. The addition of flexible fibers to granular beds of spheres increases the yield shear stresses of the beds [19], while how such addition of the fibers affects the constitutive relations of the flows remains unknown.

Machine learning (ML) techniques [65,66,67,68,69] offer a promising approach for advancing the study of granular mechanics and flow behavior by identifying complex, nonlinear relationships in experimental and simulation data—relationships that are challenging to capture using traditional methods. ML can aid in the development of predictive models by analyzing high-dimensional data, improving the accuracy of constitutive model parameter estimations [66,67], and effectively capturing the influence of fiber characteristics—such as aspect ratio and bending elasticity—on their mechanical behavior. Additionally, ML algorithms and persistent homology have been successfully applied to analyze stress transitions in granular materials [68,69], demonstrating their potential for studying fiber materials as well. Future research should explore how these advanced techniques can be more effectively utilized to address the unique mechanical challenges associated with flexible fiber materials, thus enhancing our understanding of their flow dynamics and mechanical properties.

A promising direction for future studies would be to identify the optimal fiber shapes that can effectively regulate the mechanical properties of fiber-based materials. This could be achieved through a combination of advanced numerical simulations and ML techniques, which would enable the development of predictive models that account for the complex interactions between fiber geometry and material properties. Such models would provide valuable insights into how fiber characteristics can be tailored to achieve desired mechanical behaviors in fiber-based systems.

In the current numerical studies on the two-phase flows with high concentrations of the fibers, the accuracy of unresolved calculations of the gas forces and torques exerted on the fibers are not high. In future research, the models of gas forces and torques need to be improved by more accurately considering the effects of the presence of neighboring fibers, which causes flow disturbance near the fiber of interest. In addition, high quality experimental data of the two-phase flows with the fibers are needed to validate the numerical methods.
